# Bacterial contamination of the hands of intensive care unit staff during respiratory tract care: preliminary results

**DOI:** 10.1186/2047-2994-4-S1-P246

**Published:** 2015-06-16

**Authors:** C Landelle, A De Gea-Hominal, S Touveneau, E Genevois, N Colaizzi, A Gayet-Ageron, D Scalia, V Sauvan, J Schrenzel, P Francois, J Pugin, D Pittet

**Affiliations:** 1 Infection Control Program, University of Geneva Hospitals, Geneva, Switzerland; 2 Intensive Care Unit, University of Geneva Hospitals, Geneva, Switzerland; 3 Genomic Research Laboratory, University of Geneva Hospitals, Geneva, Switzerland

## Introduction

Optimal care of the respiratory tract (RT) is critical to prevent ventilator-associated pneumonia in ICU. The dynamics of microbial cross-transmission by the hands of health care workers (HCW) during RT care is currently unknown.

## Objectives

To study the level of HCWs’ hand contamination during RT care.

## Methods

Structured observations of RT care sequences were conducted by trained external observers. At the beginning and the end of each care sequence observed, imprints of the 5 fingertips of the dominant hand, with and without gloves, were taken on blood agar plates. Bacterial colony-forming units (CFUs) were quantified after 18 hrs of incubation at 35ºC. The primary outcome was the number of CFU/plate at the end of the care sequence and before performing hand hygiene, expressed as medians and interquartile range.

## Results

A total of 207 structured observations were performed: nasal care (n=31), nasal care with fixing of the naso-gastric tube (NGT;n=31), oral care with water (n=29), oral care with chlorhexidine (CHX;n=50), fixing of respiratory tube (n=33) and endo-tracheal aspiration (n=33). Hand hygiene compliance before aseptic care was 70%. Gloves were used for 94.2% of care sequences and a gloves’ contamination >10 CFUs before care was observed in 24.2% of care sequences. When considering RT care activities with ≤10 CFUs on hands or gloves at the start of care, we observed a median of 49 [6-88], 111 [37-277], 140 [50-317], 43 [14-168], 156 [15-308], and 4 [1-18] CFU/plates for nasal care (n=23), nasal care with fixing of the NGT (n=21), oral care with water (n=23), oral care with CHX (n=33), fixing of the respiratory tube (n=21) and endo-tracheal aspiration (n=26), respectively.

## Conclusion

Among different types of RT care in intubated patients, fixing of the NGT or respiratory tube and oral care with water showed the higher levels of bacterial contamination. Oral care with CHX was associated with lower contamination levels than oral care with water. Further analyses will be conducted to model the dynamics of bacterial contamination according to the duration of care.

## Disclosure of interest

None declared.

